# Bronchial and alveolar airway nitric oxide levels in primary ciliary dyskinesia and other respiratory diseases

**DOI:** 10.1186/2046-2530-1-S1-P10

**Published:** 2012-11-16

**Authors:** W Walker, A Liew, A Harris, JS Lucas

**Affiliations:** 1 Faculty of Medicine, University of Southampton, UK; 2 University Hospitals Southampton NHS Foundation Trust, UK

## Background

Patients with primary ciliary dyskinesia (PCD) have abnormal ciliary function and low nasal nitric oxide (nNO) and fractional exhaled NO (FeNO). Nitric oxide (NO) biosynthesis is dependent on nitric oxide synthases (NOS). Cilia line the bronchial but not the alveolar epithelium. It has been hypothesised that NOS function relies on normal ciliary function and that in PCD bronchial (JNO) but not alveolar (CalvNO) NO might therefore be reduced. The aim of this study was to compare JNO and CalvNO in children with PCD, cystic fibrosis (CF), asthma and healthy subjects.

## Methods

Multiple flow rate FeNO (50, 100, 200 and 250 ml/s) and nNO measurements were performed using an NIOX® NO analyser (Aerocrine, Solna, Sweden) in children with PCD (n=14), asthma (n=18), CF (n=12) and healthy controls (n=18). JNO and CalvNO were derived using a model of pulmonary NO exchange-dynamics.

## Results

Both the mean (SD) JNO and CalvNO were significantly lower in PCD than healthy children (264 picolitres/second (pl/s) (209) vs 720 pl/s (514), p=0.024 and 1.7 parts per billion (ppb) (0.8) vs. 3.5 ppb (1.3), p=0.001 respectively.) JNO in asthmatics was found to be significantly higher than in healthy controls and CalvNO in CF children was significantly lower (2100pl/s (1935), p=0.045 and 2.5ppb (1.2), p=0.034 respectively).

## Conclusion

Children with PCD had significantly lower JNO and CalvNO compared to healthy controls. This does not support the hypothesis that NOS and ciliary function are coupled.

